# Case report: 10 years follow-up of psychosis due to Fahr’s disease complicated by a left temporal stroke

**DOI:** 10.3389/fpsyt.2023.1268982

**Published:** 2023-10-31

**Authors:** Marco De Pieri, Greta Poglia, Javier Bartolomei

**Affiliations:** Division of Adult Psychiatry, Department of Psychiatry, University Hospitals of Geneva, Geneva, Switzerland

**Keywords:** Fahr’s disease, Parkinson personality, psychosis, diagnosis, antipsychotics

## Abstract

Fahr’s disease (FD) is a rare disorder, characterized by basal ganglia calcification and presenting with movement disorders, speech impairment, cognitive deficits, and neuropsychiatric symptoms. Psychotic disorders related to FD are barely described in the literature, and knowledge is missing concerning pathophysiology, course, and management. Here, we report on the long-term follow-up of a patient who had three acute episodes of FD-psychosis characterized by bizarre delusions and behavioral disorganization, without hallucinations. Genetic and metabolic causes of FD were ruled out. In all three episodes, olanzapine monotherapy rapidly and completely resolved psychosis, without inducing metabolic syndrome and extrapyramidal symptoms. In addition to the acute decompensations, the patient presented a tame, introverted, industrious, and perfectionistic personality, which we could interpret as the “*parkinsonian personality*” described for many other basal ganglia disorders. Moreover, bizarre appearance, reduced affectivity, abulia, concrete speech, and stiff motricity in the context of a mild asymmetric extrapyramidal syndrome characterized the mental status. The cognitive profile was initially marked by executive difficulties and partial agnosia, with an IQ of 86. In the course of 10 years, the patient suffered from an ischemic stroke in the left superior temporal gyrus, which provoked a decline in memory and executive functions, without any impact on the psychiatric picture. Antiphospholipid antibody syndrome emerged as the underlying cause; thus, for the first time in the literature, an overlap of FD and antiphospholipid antibody syndrome is described here. This case report stresses once more the need for better integration of psychiatry and neurology and for the investigation of secondary causes of late-onset psychosis.

## Introduction

Fahr’s disease (FD) is a rare disorder (prevalence <1 out of 1,000,000), identified by progressive brain calcifications of internal globus pallidus, caudate, putamen, dentate nuclei, and thalamus (1, 2).

The pathogenesis of FD is incompletely understood; a calcification of small vessels’ walls and perivascular space occurs, ultimately extending to neurons. A defect in iron transport and a free radical production at the level of the blood–brain barrier are considered as pathogenetic *primum movens*. Thus, microvascular stenoses initiate a cycle of impaired blood flow, neural tissue injury, and mineral deposition (1, 3).

FD is sporadic in most cases, but it is rarely inherited in an autosomal dominant fashion due to mutations in solute carrier 20A2 (SLC20A2), platelet-derived growth factor beta (PDGF-beta), platelet-derived growth factor beta receptor (PDGFR-beta), and Xenotropic and Polytropic Retrovirus Receptor 1 (XPR) genes.

FD needs to be distinguished from Fahr’s syndrome in which basal ganglia calcifications are attributed to metabolic, mitochondrial, infectious, toxic, or traumatic causes (1, 4).

FD’s typical age of onset is in the 3rd to 5th decade of life, being extrapyramidal syndrome the most common presentation. Affected individuals display parkinsonism, ataxia, dystonia, seizures, myoclonus, speech impairment, cerebellar dysfunction, orofacial dyskinesias, and chorea (1, 2, 4). Cerebrovascular disorders were described in many cases of FD (5, 6). Neuropsychiatric symptoms are common in FD, including major cognitive impairment, frontal lobe syndrome, mania, depression, and personality change (4, 7). So far, few cases of FD-psychosis have been described (8–14).

Treatment for FD is usually symptomatic, while no disease-modifying measures exist to limit the progression of brain calcification (1, 2).

Even if FD-psychosis is a known phenomenon, in-depth knowledge of clinical features, comorbidities, course, and treatment is lacking. For the first time in the literature, we describe a long-term follow-up of a patient with non-hereditary idiopathic FD, developing multiple psychotic episodes in the course. Moreover, the patient represents the first case in the literature of antiphospholipid antibody syndrome comorbid with FD, leading to a left-superior parietal stroke.

## Case report

Mr. B is a Caucasian man, who was born in 1961. No pregnancy and/or perinatal problems are known nor is there a delay in the early development. Since the age of 8 years, he was admitted to a special school due to unspecified learning difficulties, corresponding to a 2 years delay compared to the norm. Mr. B was then able to move to an ordinary school at the age of 14 years, but he did not obtain the graduation. After a practical education, he worked as a store manager for 13 years and then, until 2008, as a delivery boy for his religious community. His only relevant medical antecedent was cranial trauma with peri-circumstantial amnesia at the age of 8 years; he has no family history of movement disorders or psychosis, but his only brother has a slight intellectual disability and is institutionalized.

In 2012, without any provoking factor, Mr. B suffered from a psychotic episode characterized by bizarre mystic and persecutory delusions accompanied by congruent hallucinations, requiring hospitalization due to psychomotor agitation and lack of insight. An idiopathic brief psychotic episode was diagnosed, and after administration of olanzapine 10 mg, symptoms got completely remitted in 7 days. The same treatment was continued for relapse prevention.

After 2 years, following olanzapine withdrawal for 4 months in accordance with the psychiatrist, a novel brief psychotic episode ensued. The patient was arrested by the police on the street while walking barefoot; upon interrogation, he explained that this way he would have found a wife the same day, a delusion that was also mixed with some of the mystic contents known from the previous episode. Again, a 7 days hospitalization and the resumption of olanzapine 10 mg made full remission.

After discharge, he was addressed for a neurological assessment: a predominant right hypokinetic rigid extrapyramidal syndrome was observed, attributed mostly to FD, and to olanzapine treatment only to a lesser extent. A brain MRI displayed abnormal calcium deposits at the level of the basal ganglia, especially of the pulvinar, leading to a diagnosis of FD. Magnetic resonance angiography and venography excluded intracranial large-vessel stenosis, aneurysm, vascular malformation, or dural sinus thrombosis. The contrast-enhanced MRI excluded abnormal parenchymal or leptomeningeal enhancement. An endocrinological evaluation ruled out hyperparathyroidism and other causes of a phospho-calcic metabolism imbalance since repeated blood dosages of total calcium, ionized calcium, phosphates, parathyroid hormone, and 25-hydroxy-vitamin D showed no abnormalities. After this comprehensive neurological and endocrinological assessment, the two psychotic episodes were evaluated as a form of FD-psychosis.

Extensive genetic testing (SLC20A2, PDGF-beta, PDGFR-beta, and XPR) was realized, but no pathogenetic mutation was found in homo- or heterozygosis.

Because of the subjective difficulties in reading, writing, and calculating a neuropsychological realized. It resulted overall normal, even if some executive difficulties and a partial agnosia emerged, with an IQ of 86 (see Table 1 for the summary of testing).

**Table 1:** Summary of neuropsychological deficits at multiple time points.

Cognitive function	Test	Results	Interpretation
*24.04.2014*			
Mental flexibility	Color trail test; WAIS-III subtest code	NA	Lower limit of normality
Attention	Color trail test; WAIS-III subtest code	NA	Moderate impairment
Verbal IQ	WAIS-III	Total score 86 pts, performance score 84 pts	Lower limit of normality
*19.01.2016*			
Global assessment	MoCA test	Total score 24/30 pts (−1 pt. mini trail, −1 pts serial subtractions, −1 pt. fluency, −3 pts delayed recall)	Lower limit of normality
Executive functions	BREF scale	Total score 16/18 pts (− 1 pt. fluency, − 1 pt. Go/noGo)	Lower limit of normality
*11.01.2017*			
Global assessment	MoCA test	Total score 24/30 pts (−1 pt. mini trail, −1 pts serial subtractions, −1 pt. fluency, −3 pts delayed recall)	Lower limit of normality
Executive functions	BREF scale	Total score 16/18 pts (− 1 pt. fluency, − 1 pt. Go/noGo)	Lower limit of normality
*19.12.2018*			
Global assessment	MoCA test	Total score 25/30 pts (−1 pt. clock, −1 pt. fluency, −2 pt. free recall, −1 pt. orientation)	Normal
*10.01.2020*			
Global assessment	MoCA test	Total score 26/30 pts (−1 pt. clock, −1 pt. fluency, −1 pt. free recall, −1 pt. orientation)	Normal
*25.01.2022*			
Verbal fluency	Animal categories	23 pts	Pathological
Phonemic fluency	Words starting with “P”	19 pts	Lower limit of normality
Mental flexibility	Color trail test	1st part 85″, 2nd part 210”	Pathological
Inhibition	Stroop Victoria test	24”	Pathological
Verbal memory	Immediate recall	14 pts	Lower limit of normality
Episodic memory	Doors test	Part A 9 pts, part B 5 pts	Lower limit of normality
Attention	WAIS-III subtest code	35 pts	Pathological
Emotion recognition	Mini-SEA test	Fear not recognized in 4/5 attempts	Pathological
*15.02.2022*			
Global assessment	MoCA test	23/30 pts (−3 pts executive functions, −1 pt. verbal fluency, −1 pt. abstraction, −2 pts delayed recall)	Pathological

Pt/pts, point/s; NA, not available; WAIS, Wechsler adults inventory scale; IQ, intelligence quotient; MoCA, Montreal cognitive assessment; BREF, rapid battery for the frontal efficiency; Mini-SEA, mini-social cognition and emotional assessment.

In 2018, olanzapine was interrupted again in accordance with the psychiatrist, considering the 4 years disease-free interval and the risk of metabolic syndrome and of a worsening of extrapyramidal symptoms with this medication. After 3 weeks of drug withdrawal, Mr. B developed an acute confusional state and a seizure due to hyponatremia (124 nM) secondary to potomania. Once the ion imbalance was resolved, mental status turned out to be characterized by an incoherent and disorganized thought process, and thought content was occupied by the delusional idea of having killed his brother from a distance, which generated feelings of guilt and shame. Moreover, the obsessional fear that he or someone else was going to die emerged for some days. Once more, olanzapine reintroduction at a dosage of 15 mg resolved the acute episode. After a month, a maintenance dose of 10 mg was left and is still effectively taken.

**Figure 1:**
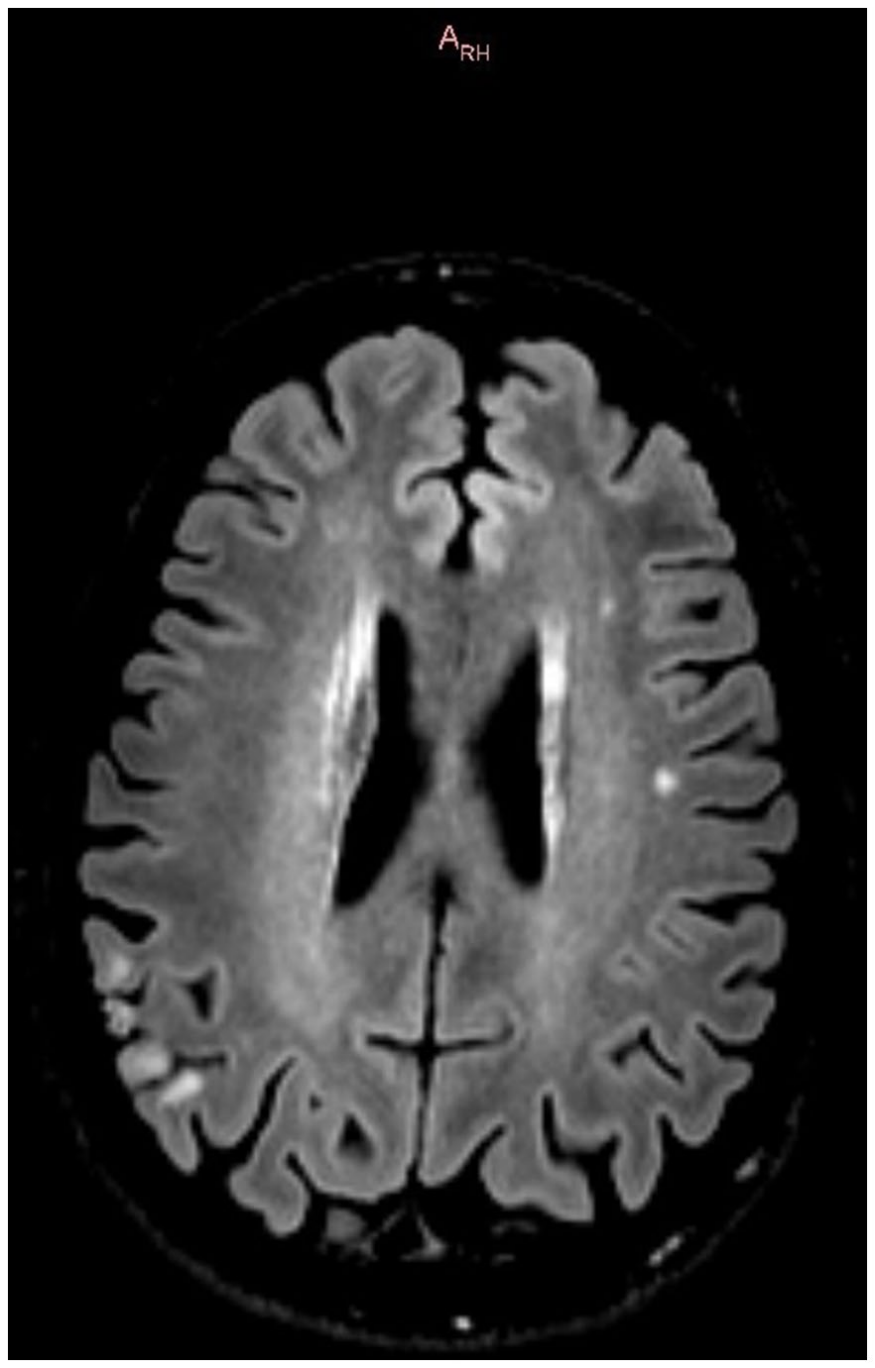
3T brain MRI displaying basal ganglia calcification and left temporal stroke, 15.11.2023.

In January 2022, the patient complained of a subjective decline in memory and executive functions, mirrored by a Montreal Cognitive Assessment test (MoCA) score drop from 26 to 23 points (see Table 1). A novel brain MRI revealed an ischemic stroke in the left superior temporal gyrus (see Figure 1), without any other relevant difference compared to previous MRIs. A duplex of the sovra-aortic vessels and a long-term Holter exam of the cardiac rhythm revealed no abnormalities; meanwhile, the echocardiography showed a right–left shunt of moderate severity, confirmed by means of a transcranial duplex. The laboratory examination showed the positivity of the B2GP1 antibodies (53.3 UI/L at time 0, 61.6 UI/L after 6 weeks), leading to a diagnosis of antiphospholipid antibody syndrome. Anticoagulation with warfarin was introduced and is still ongoing. A comprehensive neuropsychological evaluation showed a general slowing and deficits of selective attention, verbal episodic memory, recognition of fear, and verbal autoactivation.

The last brain MRI in 2022 displayed stability of the basal ganglia calcifications and of the ischemic lesion but a hippocampal atrophy (class MTA 0 bilaterally) and a microvascular leukoencephalopathy (Fazekas 1 grade).

Nowadays, Mr. B is independent in the activities of daily living and in self-care; he lives alone in an apartment, and he benefits from a disability monthly paycheck and works part-time in a laundry. He displays a poor relational and intimate life: he is not in a couple relationship, has a social life restricted to a religious community, and his sister remains his main support figure.

The patient’s personality is tame and naïve, characterized by interpersonal inhibition and social withdrawal and also by obsessive traits such as perfectionism, ritualism, over-conscious behavior, and love for habits. Currently, Mr. B presents no positive psychotic symptoms and behavioral abnormalities, but his appearance is bizarre and aloof; he gives the examiner a sense of strangeness and coldness in a context of reduced affectivity and stiff motricity. Mr. B always reports feeling good but displays apathy, abulia, and anhedonia instead, and his speech is concrete and oversimplified. From the motor point of view, Mr. B presents an asymmetric bradykinetic-amimic extrapyramidal syndrome and bucco-facial dyskinesias, as detailed in Table 2.

**Table 2:** MDS-UPDRS III, 05.08.2022.

ITEM	Left	Right
3.1 Speech	0/4	
3.2 Facial expression	2/4	
3.3 Rigidity	2/4	2/4
3.4 Finger tapping	2/4	1/4
3.5 Hand movements	1/4	1/4
3.6 Pronation-supination movements of hands	2/4	2/4
3.7 Toe tapping	1/4	1/4
3.8 Leg agility	1/4	1/4
3.9 Arising from chair	0/4	0/4
3.10 Gait	0/4	0/4
3.11 Freezing of gait	0/4	0/4
3.12 Postural stability	0/4	0/4
3.13 Posture	0/4	0/4
3.14 Global spontaneity of movement	0/4	0/4
3.15 Postural tremor of hands	0/4	0/4
3.16 Kinetic tremor of hands	0/4	0/4
3.17 Rest tremor amplitude	0/4	0/4
3.18 Constancy of rest tremor	0/4	0/4
TOTAL	19/132	

MDS-UPDRS III, Movement Disorder Society-Unified Parkinson’s Disease Rating Scale, 3rd section; cutoff scores to define mild/moderate and moderate/severe levels are 32/33 and 58/59, respectively.

Beyond the three psychotic episodes, psychiatric status and personality profile remained remarkably steady, with no meaningful variations through the years; after each psychotic episode, Mr. B was able to recover his previous level of functioning, without personal and cognitive impairment nor residual psychotic symptoms; a decline was only due to the ischemic stroke.

The patient persistently showed good insight, with a full understanding of the motor symptoms of FD and of the three episodes of FD-psychosis. Accordingly, he has been taking olanzapine for a long time with perfect adherence and categorically refused a novel attempt at withdrawal or a switch to a different medication.

## Discussion

FD is a rare neurological condition having multiple dimensions; FD-psychosis is one of them, whose pathophysiology, course, and treatment remain poorly understood. The present case is unique in terms of follow-up length. Moreover, attention should be given to the overlap with a left superior temporal stroke resulting from an antiphospholipid antibodies syndrome and to an episode of psychogenic polydipsia. This latter is an impulsive phenomenon unknown in FD but possibly linked to basal ganglia dysfunction (15).

Mr. B presented a “parkinsonian personality,” arising in the context of a basal ganglia disorder (e.g., Parkinson’s disease) and defined as a compulsive, industrious, introverted, morally rigid, cautious, punctual, quiet, and inflexible character, with low novelty seeking (16). Psychotic episodes of Mr. B were not in line with this personality structure but similar to schizophrenic delusions, normally arising on the grounds of a paranoid or schizotypal personality (17).

In fact, acute psychotic symptoms consisted of the delusion of persecutory type, bizarre and disorganized behavior, and in a lesser component thought process impairment. These features were in line with other case reports (8, 18), but Mr. B differed in the absence of hallucinations (10, 12–14, 18) and of obsessive-compulsive symptoms (10). The onset of psychosis was late (51 years), in line with the typical age of onset of FD (1, 2, 4) and with some cases of FD-psychosis (8–10, 13). On the contrary, other cases of FD-psychosis had an early onset (11, 12, 14). Mr. B was determined as a sporadic case of FD-psychosis after an extensive search for genetic causes of the disorder and an investigation into affected relatives. Quite the opposite, any of the other cases of FD-psychosis in the literature underwent genetic testing, and only for one of them, a familial nature was acknowledged (14).

We observed a complete remission of Mr. B’s acute psychotic episodes and a return to the same baseline level of functioning, without decline, which differentiates FD-psychosis from schizophrenia. In fact, each acute episode of schizophrenic psychosis takes a toll in terms of cognitive, social, and personal functioning, and often residual psychotic symptoms persist (19).

The concomitance of ischemic stroke with FD has already been described (5, 6, 20) and linked to genetic mutations related to FD in two cases (21, 22). Calcification in the walls of small vessels has been implicated as the potential cause (6). In addition to this possible mechanism, we describe for the first time in the literature the comorbidity of FD with a phospholipid antibodies syndrome, interpreted as the cause of stroke. The parietal ictus had no consequences on psychopathology but only on cognitive functioning. This finding is surprising considering the role of the area in behavioral disorganization (23) and in the pathogenesis of psychosis (24).

In the 10 years clinical history of Mr. B, treatment was specific for FD-psychosis, without medication to address other dimensions of FD. Response to olanzapine was complete and sustained, with no induction of metabolic syndrome nor of a significant movement disorder; in fact, the patient presents a mild extrapyramidal syndrome, mostly attributed to FD. Olanzapine withdrawal led to multiple psychosis relapses, proving that long-term medication was needed; the ischemic stroke did not pose the need for a change in this respect. The choice of olanzapine was motivated by the concern that an antipsychotic with a stronger anti-dopaminergic effect would have worsened the extrapyramidal syndrome. Our choice was in line with a previous case that was effectively treated with olanzapine alone (18) or combined with fluoxetine to address concomitant obsessive symptoms (10). However, other cases treated with low-dose risperidone (12, 13), with risperidone plus oxcarbazepine (11), with low-dose haloperidol (9), or with risperidone plus haloperidol (8) that had an equally positive outcome in the short-term, without developing a movement disorder. These findings indicate that all antipsychotics could be rapidly effective in inducing remission of FD-psychosis. Clozapine is possibly a valuable therapeutic option, often used for psychosis in Parkinson’s disease and other basal ganglia disorders due to the low impact on the extrapyramidal system (25). However, the high potential for sedation and metabolic syndrome relegates its use to treatment-resistant schizophrenia (26).

Our case report presents many strengths, such as the length of the observation period, the multidisciplinary follow-up, the availability of neuropsychological and neuroradiological data, and the careful mental status evaluation at multiple time points. The overlap between FD and antiphospholipid antibodies syndrome is unprecedented. However, some limitations can be found: the diagnosis of FD was delayed and posed only after the second psychotic episode. Second, in-depth neuropsychological testing was realized only twice, while the cognitive follow-up was mainly based on screening instruments such as the Montreal Cognitive Assessment (MoCA) and the rapid battery for the frontal efficiency (BREF) scales. Moreover, concerning the first neurocognitive evaluation, we only had a qualitative evaluation of the outcome of many tests, with the raw results not available in the clinical records. Last, a formal evaluation for personality disorders was missing.

In conclusion, our report underlines the necessity of excluding organic causes in front of an acute psychotic onset, especially at an age range atypical for a first episode of schizophrenia. Imaging and laboratory exams should be integrated into psychiatry clinical practice in order to diagnose organic mental disorders. FD-psychosis remains a poorly studied phenomenon, deserving further investigation to reach an in-depth knowledge about pathophysiology and clinical management.

## Data availability statement

The raw data supporting the conclusions of this article will be made available by the authors, without undue reservation.

## Ethics statement

Written informed consent was obtained from the individual(s) for the publication of any potentially identifiable images or data included in this article. Written informed consent was obtained from the participant/patient(s) for the publication of this case report.

## Author contributions

MP: Conceptualization, Methodology, Writing – original draft, Writing – review & editing. GP: Supervision, Validation, Writing – review & editing. JB: Supervision, Validation, Writing – review & editing.
